# Mother-infant interaction assessment at discharge and at 6 months in a French cohort of infants born very preterm: The OLIMPE study

**DOI:** 10.1371/journal.pone.0188942

**Published:** 2017-12-07

**Authors:** Gilles Cambonie, Jean-Baptiste Muller, Virginie Ehlinger, Joël Roy, Antoine Guédeney, Cécile Lebeaux, Monique Kaminski, Corine Alberge, Sophie Denizot, Pierre-Yves Ancel, Catherine Arnaud, on behalf of the OLIMPE study writing group

**Affiliations:** 1 Department of Neonatal Medicine, Arnaud de Villeneuve University Hospital, Montpellier, France; 2 Department of Neonatal Medicine, Women’s and Children’s University Hospital, Nantes, France; 3 UMR 1027 INSERM, University Paul Sabatier Toulouse III, Toulouse, France; 4 Child and Adolescent Psychiatry Unit, Nîmes University Hospital, Nîmes, France; 5 Child and Adolescent Psychiatry Unit, Bichat-Claude Bernard University Hospital, Paris, France; 6 Inserm UMR 1153 Obstetrical, Perinatal and Paediatric Epidemiology Research Team (Epopé), Centre for Epidemiology and Statistics Sorbonne Paris Cité, DHU Risks in Pregnancy, Paris Descartes University, Paris, France; 7 Department of Neonatal Medicine, Children's University Hospital, Toulouse, France; King Abdulaziz University Hospital, SAUDI ARABIA

## Abstract

**Objectives:**

The principal aim was to investigate the feasibility of assessing mother-infant interactions at discharge and at 6 months infant corrected age in singletons born before 32 weeks of gestation. The secondary aims were to describe these interactions and their disorders, explore the association between maternal emotional state and the interactions, and assess the relationship between disordered interactions and infant social withdrawal behaviour.

**Methods:**

OLIMPE is an ancillary study of the population-based study EPIPAGE 2, which recruited preterm neonates in France in 2011. 163 dyads participated at discharge and 148 at 6 months. Interactions were observed with the Attachment During Stress (ADS) scale, which includes two behavioural subscales, for the mother (m-ADS) and her infant (i-ADS). Two professionals independently completed the ADS scales for one third of the observations. Maternal emotional state was assessed using self-administered questionnaires of depression, anxiety, and stress. Infant’s social withdrawal behaviour at 6 months was measured by the Alarm Distress Baby scale.

**Results:**

At discharge, 15.3% of the m-ADS scales and 43.3% of the i-ADS scales had at least one unobserved component. At 6 months, all items on both scales were noticeable in >90% of the dyads. Reliability, estimated by the kappa coefficient, ranged between 0.39 and 0.76 at discharge, and between 0.21 and 0.69 at 6 months. Disordered interactions were indicated on 48.6% of the m-ADS scales and 36.5% of the i-ADS scales at discharge. At 6 months, these rates were 32.6% and 26.0%. Disordered interactions at 6 months were associated with identified disorder at discharge. Insecure infant attachment was not influenced by maternal mental health but was strongly associated with infant social withdrawal behaviour.

**Conclusions:**

The ADS scale can be used to screen for early interaction disorders after premature birth and may help to target dyads that would most benefit from early intervention.

## Introduction

Improvements in perinatal care, including technical practices in neonatal intensive care units (NICUs) and management of high-risk pregnancies, have led to an increase in the survival rate of very preterm infants [1]. However, preterm birth is associated with a high risk of long-term sequelae, such as neuromotor disabilities, cognitive impairment, emotional disturbance and behavioural problems [2–3]. Several risk factors have been identified in the genesis of these morbidities, including somatic factors like neonatal cerebral lesions, bronchopulmonary dysplasia, sepsis, and protein and energy undernutrition [4–7], and environmental factors like the family’s sociocultural level and the parental affective state [8–11].

Parental emotions and attitudes following a preterm birth may affect the quality of the early parent-infant relationship and possibly the child’s later competencies and development [12–15]. Indeed, prematurity is a risk situation that may well disrupt parent-infant interactions. Admission to the NICU means intensive technical care and a long stay in an incubator, which limits parental proximity to their newborn. These risks have been taken into account in the developmental care programs now implemented in the NICUs of many countries, giving a central place to parental competencies and well-being through a family-centred approach [16]. The physiological characteristics of preterm infants also pose a risk for interactions. During the neonatal period, preterm infants may display signs of weaker or shorter attentional behaviour, more fragile wakefulness, and less responsivity to caregivers than full-term infants [17]. In turn, the stress and traumatism of the preterm birth may alter the mother's sensitivity and availability, although these elements are crucial for the development of mother-infant bonding [18]. Alterations in the quality of the mother-infant interaction and maternal attachment representations have been reported, indicating a more mother-controlling dyadic pattern of interaction than after a term birth [13, 19, 20]. This pattern has been associated with infant’s behavioural symptoms, eating difficulties, and lower interest or abilities for social communication in the first two years of life [13]. Another potential consequence of a disorganized maternal caregiving system is social withdrawal behaviour, which is more frequent in children born preterm [21]. The early identification of maternal or parental interaction patterns that signal risk for infant development could prompt supportive intervention at a very early age: while the infant is still in the NICU and/or shortly after discharge to home.

Few observational-assessment tools are available to evaluate early mother-infant interactions, particularly in the context of premature birth [22]. Massie and Campbell developed the Attachment During Stress (ADS) scale to observe mother and child behaviours during paediatric examinations or other stressful situations [23]. The scale was particularly designed to detect insecure attachment behaviour. Studies have reported on its use in clinical paediatric settings as part of a Chilean attachment and intervention program [24] and in the investigation of mother-infant interactions with adoptive mothers or HIV-positive women [25]. This scale is suitable from birth to 18 months, but no study has yet established its feasibility in the context of a discharge examination of a very preterm infant.

EPIPAGE 2 is a cohort study of all preterm infants born between 22 and 31 completed weeks of gestation in 2011 in France, covering a period of 8 months [26]. The OLIMPE project (Observation LIen Mère-enfant PrématurE, observation of mother-premature infant bonding) provides a window for more detailed exploration of mother-infant interactions in a subsample of the French EPIPAGE 2 cohort. To this end, a multidisciplinary team of perinatal professionals was simultaneously trained to the same standards for observing interactions and assessing social withdrawal behaviour in two different periods: when the child was discharged from the NICU and at 6 months corrected age (chronological age plus prematurity).

The primary objective of this study was to determine the feasibility of using the ADS scale for very preterm neonates, both at the time of discharge to home and at 6 months corrected age. The secondary objectives were to (i) describe the mother-infant interactions and any disorders at discharge and 6 months, (ii) explore the association between maternal emotional state—including depression, anxiety, and stress—and these interactions, and (iii) assess the relationship between the disordered interactions and infant social withdrawal behaviour. An exploratory objective included the relationship between disordered interactions and family-centred care, evaluated by the centres’ policies.

## Materials and methods

### EPIPAGE 2 cohort

The EPIPAGE 2 study is a French prospective multicentre observational cohort study designed to describe short- and long-term outcomes in preterm infants and their families and to identify early predictors of health and developmental problems. Detailed information about the study is available elsewhere [26]. Additional investigation of the early mother-infant relationship was offered to the families recruited in the 12 volunteer centres via the OLIMPE project. In this project, data on mother-child interactions, the infant’s social withdrawal behaviours, and the mother’s mental health were collected at discharge and at 6 months corrected age.

As required by French law and regulations, the OLIMPE study was approved by the national data protection authority (Commission Nationale de l’Informatique et des Libertés: CNIL n°911009) and by the Committee for the Protection of People Participating in Biomedical Research (CPP: Comité de protection des Personnes, CPP C11-04).

### Study population

We contacted all the mothers enrolled in the EPIPAGE 2 cohort study with singletons born before 32 weeks of gestation, hospitalized in one of the 12 participating NICUs from nine French regions, and alive at 36 weeks corrected age. Recruitment and data collection occurred only after families had received information on the OLIMPE project and agreed to participate in the study.

The exclusion criteria were as follows: severe neurological complications of prematurity (cystic periventricular leukomalacia and grades 3 and 4 severe intraventricular haemorrhage), congenital malformations, mother’s age under 18 years, and mother suffering from a psychiatric disorder and/or drug/alcohol abuse or with difficulty in speaking and understanding French.

### Data collection

According to the EPIPAGE 2 protocol [26], data were collected in the maternity and neonatal units, extracted from medical records and completed by questions to the obstetrical and neonatal teams. The data included family, socioeconomic, pregnancy and newborn characteristics. The NICUs’ policies regarding family-centred care were addressed in EPIPAGE 2 by structured NICU questionnaires that focused specifically on two types of neurodevelopmental care: skin-to-skin (kangaroo) care (KC) and breastfeeding (BF) [27]. Each mother also completed a self-administered questionnaire assessing her mental health and the management of her infant in the neonatal unit, just before the infant’s discharge.

### Scales and scores

#### ADS

Baseline mother-child interaction was assessed at the infant’s visit before discharge from the NICU, using the ADS scale of mother-infant attachment indicators during stress [23]. The ADS scale is designed to provide indicators of mother/father attachment to infants and toddlers from birth to 18 months. The infant subscale (i-ADS) measures the infant’s behaviour during a stressful event, whereas the maternal subscale (m-ADS) measures the mother’s response to the infant’s stress. Each subscale explores seven basic attachment components: gazing, vocalizing, touching (a) (i.e. touches or reaches towards mother), touching (b) (i.e. pulls away from mother’s touch), maternal holding, affect and physical proximity. Each item is scored on a 5-point scale based on frequency and intensity during the observation period. A score of 1 indicates very avoidant behaviour, 2 rather avoidant behaviour, 3 and 4 typical attachment behaviour (i.e. using the attachment figure as a secure base), and 5 clinging and unusually strong reaction to stress. At 6 months corrected age, the same observational ADS scale was used.

The ADS scales were scored immediately after a brief standardized observation of mother and child reactions during a routine paediatric examination. All observers (at least 2 per centre) were trained at the same time to score these mother-infant interactions. The training was based on videotape sessions provided by a psychiatrist specialized in perinatal care, and it was conducted before the OLIMPE project began. In nearly one third of the observations, two trained professionals were simultaneously present and scored the ADS scales independently. On each subscale (m-ADS and i-ADS), an interaction disorder was defined by one insecure-resistant behaviour (i.e. a score of 5 for at least 1 item of the ADS scale), one insecure-avoidant behaviour (i.e. a score of 1 for at least 1 item), or two rather avoidant behaviours (i.e. a score of 2 for at least 2 basic attachment components).

#### ADBB

At 6 months corrected age, infant social withdrawal behaviour was assessed with the Alarm Distress Baby (ADBB) scale. The scale has eight items, each rated 0–4: facial expression, eye contact, general level of activity, self-stimulating gestures, vocalizations, response to stimulation, ability to engage in a relationship, and attraction. The ADBB scale was developed by Guedeney and Fermanian [28] to assess relational withdrawal in infants 2–24 months old during a routine physical examination. A score of 5 or over is assumed to detect those infants with unusually low social behaviour [29].

#### CES-D

Mothers' depressive symptoms were evaluated at discharge and at 6 months corrected age using the Center for Epidemiologic Studies Depression Scale (CES-D). The CES-D is a well validated and commonly used self-administered screening test for depression, originally developed by Radloff [30]. It comprises 20 questions that measure depressive feelings and behaviour during the past week, each question being scored from 0 (rarely or none of the time, <1 day) to 3 (most or all of the time, 5–7 days). The total score ranges from 0 to 60 and the higher the score, the more severe the depressive symptoms. A total score of 16–22 indicates possible depression, whereas a score equal to or above 23 denotes probable depression.

#### STAI

The State-Trait Anxiety Inventory for adults (STAI), developed by Spielberger et al. and validated in French in 1993, was administered to mothers at discharge and at 6 months corrected age. The STAI explores feelings of apprehension, tension, nervousness and worry. It has 20 items for assessing state anxiety (STAI-State) and 20 for trait anxiety (STAI-Trait). All items are rated on a 4-point scale (from “almost never” to “almost always”). A score of 40 or higher indicates significant anxiety [31].

#### mPPQ

Traumatic memories about the birth at 6 months corrected age were assessed using the modified Perinatal Posttraumatic Stress Disorder Questionnaire (mPPQ), a 14-item instrument especially designed for parents of high-risk infants [32]. In the modified version, mothers were asked to provide responses about symptoms that had appeared since the birth, using a 5-point Likert scale scored 0 (“never”) to 4 (“often/more than 1 month”). The posttraumatic reaction index corresponds to the sum of the responses, with the total possible score on the mPPQ ranging from 0 to 56. A score of 19 or higher, which identifies a high risk of trauma in mothers, was used [33].

### Statistical analysis

Data were analysed using STATA SE version 14 (STATA Corp., College Station, TX). P-values <0.05 were considered statistically significant. Survey weights were used to account for the difference in sampling periods, the enrolment period being longer for more premature births [26]. All analyses were weighted, unless otherwise specified.

Maternal characteristics at delivery, including demographic information, pregnancy and delivery characteristics, as well as neonatal characteristics, were described. Percentages and their 95% confidence intervals (95% CI) are presented for categorical variables; means with standard deviations (SD) are presented for continuous variables. Maternal and neonatal characteristics of the dyads were compared with those of the eligible but non-included dyads using Chi-square tests for categorical variables and the adjusted Wald test for means comparison for continuous variables. These characteristics were also compared between mother-infant dyads included and followed up at 6 months corrected age and included dyads that did not participate in the OLIMPE study at 6 months corrected age.

The feasibility of the ADS scale was assessed by estimating the rates of unobserved items for the i-ADS and m-ADS subscales on the sample, and interrater reliability was evaluated for the subsample of dyads that were observed independently by two professionals. Interrater agreement was measured for each i-ADS and m-ADS item using the percentage of observed agreement and Cohen’s linearly weighted kappa coefficient. Kappa coefficient and its 95% CI were estimated using jackknife method [34], but ignoring survey weights.

Relationships between disordered interaction at discharge and 6 months based on ADS infant and mother subscales were assessed using Pearson’s Chi-square tests and McNemar’s test for paired data.

Bivariate analyses were conducted to examine whether maternal emotional state, infant behaviour, and family-centred care were associated with disordered mother-infant interactions at discharge and 6 months corrected age. The results are presented as percentages and their 95% CI. Chi-square tests and adjusted Wald test for means comparison were used to compare the groups.

## Results

### Maternal and neonatal population

From the 12 participating NICUs, 332 neonates were eligible. Among them, 158 (48%) were not included because of (i) the unavailability of a trained professional to observe the dyad just before discharge in 149 cases, (ii) a lack of parental consent in 4 cases, and (iii) unknown reason in 5. One hundred and seventy-four parents agreed to participate in the study. The ADS scores were available for 163 mother-infant dyads at discharge, 148 at 6 months, and 136 at both times. Tables 1 and 2 present the maternal and neonatal characteristics of the eligible sample, the included sample, and the included sample still participating in the OLIMPE study at 6 months corrected age. The proportion of missing data on maternal and neonatal characteristics in the included sample was less than 10% in more than 90% of the variables. Compared with the non-included population, OLIMPE mothers were older, had been hospitalized less often during pregnancy, and had less in-utero transfer. Depressive symptoms and trait anxiety were also less frequent at discharge. Compared with the non-included population, OLIMPE neonates required a shorter duration of invasive mechanical ventilation and had lower rates of severe bronchopulmonary dysplasia and abnormal auditory screening results. They were more frequently supported with KC and an individual developmental care program and were also more often breastfed with direct nipple sucking.

**Table 1:** Maternal characteristics in the eligible OLIMPE study population, the included population and the included population still participating at 6 months corrected age.

	Eligible	Included	Included and followed-up at 6 months corrected age
	(N = 332)	(N = 174)	(n = 148)
**Social characteristics**			
Age (years)	29.9 (5.8)	30.5 (5.6)*	30.7 (5.6)
Born in France	84.6 (80.1–88.2)	85.3 (79.0–89.9)	85.8 (79.0–90.7)
Social security^1^	91.0 (87.2–93.7)	91.0 (85.6–94.5)	92.3 (86.5–95.7)
Living in a couple	90.8 (87.0–93.6)	91.4 (85.9–94.9)	91.5 (85.5–95.1)
Unemployment^2^	5.5 (3.3–9.0)	4.1 (1.8–8.8)	3.9 (1.6–9.0)
**Maternal treatment**			
Chronic disease	29.3 (24.5–34.5)	26.7 (20.6–33.9)	27.0 (20.4–34.9)
Antidepressant antecedents	1.8 (0.8–4.0)	1.2 (0.3–4.7)	0.7 (0.1–4.9)
Infertility treatment	10.5 (7.5–14.4)	12.2 (8.1–18.1)	12.1 (7.7–18.5)
**Pregnancy**			
Primiparity	37.2 (32.1–42.6)	39.3 (32.3–46.8)	39.0 (31.4–47.2)
Adequate follow-up	92.6 (89.1–95.1)	93.5 (88.5–96.4)	93.8 (88.4–96.8)
Hospitalization	28.6 (23.9–33.9)	23.6 (17.8–30.5)*	24.2 (17.9–31.9)
In-utero transfer	50.0 (44.6–55.4)	41.4 (34.2–48.9)***	41.6 (33.9–49.8)
Complication			
PROM^3^ ± IUI^4^	22.2 (18.0–27.1)	22.0 (16.4–29.0)	22.9 (16.7–30.5)
Preclampsia	9.3 (6.5–13.1)	9.3 (5.7–14.8)	10.1 (6.0–16.4)
PL^5^	68.5 (63.1–73.4)	68.7 (61.2–75.3)	67.0 (58.8–74.3)
**Delivery**			
Spontaneous preterm delivery	41.4 (36.1–46.8)	43.3 (36.0–50.9)	43.2 (35.3–51.5)
Mode of delivery			
Vaginal	27.4 (22.8–32.4)	26.0 (19.9–33.0)	25.8 (19.3–33.5)
Instrumental	3.6 (2.1–6.3)	3.5 (1.5–7.5)	4.0 (1.8–8.8)
Caesarean section	69.0 (63.8–73.8)	70.6 (63.3–76.9)	70.2 (62.3–77.1)
Anaesthesia			
Regional	65.4 (60.1–70.4)	69.3 (62.0–75.7)	67.5 (59.5–74.7)
General	19.2 (15.3–23.9)	18.2 (13.1–24.7)	19.6 (13.9–27.0)
Absence	15.4 (11.8–19.7)	12.5 (8.4–18.4)	12.8 (8.3–19.3)
**Mental health at discharge**			
Depressive symptoms (CES-D)^6^		**	†
Not at risk (score <16)	54.5 (48.3–60.6)	61.5 (53.5–68.9)	65.0 (56.4–72.7)
Possible (score 16–22)	19.3 (14.9–24.7)	18.6 (13.2–25.6)	18.0 (12.3–25.6)
Probable (score ≥23)	26.2 (21.1–32.0)	19.9 (14.3–27.0)	17.0 (11.5–24.4)
STAI^7^-State >40	26.3 (21.2–32.1)	25.1 (18.8–32.6)	22.1 (15.8–30.0)†
STAI^8^-Trait >40	38.1 (32.3–44.2)	32.7 (25.7–40.4)*	27.5 (20.5–35.7)†††

Data are weighted to take into account the different inclusion periods.

Data are means (standard deviation) or % (95% CI).

^1^Usually national insurance;

^2^Unemployment is for mother and father;

^3^PROM: premature rupture of membranes;

^4^IUI: intrauterine infection;

^5^PL: other causes of preterm labour;

^6^CES-D: Center for Epidemiologic Studies Depression Scale;

^7^STAI: State-Trait Anxiety Inventory for adults, STAI-State: respondent feelings "right now";

^8^STAI-Trait: respondent feelings "generally".

*p<0.05,

**p<0.01,

***p≤0.001 included *vs* non-included.

^†^p<0.05,

^†††^p≤0.001 included and followed up at 6 months *vs* included but not followed up at 6 months corrected age.

**Table 2:** Neonatal characteristics in the eligible OLIMPE study population, the included population and the included population still participating at 6 months corrected age.

	Eligible	Included	Included and followed-up at 6 months corrected age
	(N = 332)	(N = 174)	(n = 148)
**Characteristics at birth**			
Immediate postnatal transfer^1^	9.4 (6.7–13.1)	6.5 (3.6–11.4)	6.3 (3.3–11.7)
Gestational age			
24–26 weeks	12.4 (9.5–15.9)	9.7 (6.4–14.4)	9.3 (5.9–14.5)
27–31 weeks	87.6 (84.1–90.5)	90.3 (85.6–93.6)	90.7 (85.5–94.1)
Gestational age, weeks	28.9 (1.8)	29.1 (1.8)	29.1 (1.7)
Birthweight, g	1199 (353)	1194 (329)	1198 (332)
Growth retardation, according to customized standard French curves [35]	39.0 (33.6–44.7)	40.0 (32.6–47.9)	41.5 (33.4–50.0)
Gender, female	47.8 (42.5–53.3)	47.6 (40.2–55.1)	49.3 (41.2–57.4)
**Medical complications**			
HMD^2^ requiring surfactant	50.8 (45.2–56.3)	47.1 (39.7–54.6)	47.6 (39.5–55.9)
Invasive mechanical ventilation	67.5 (62.1–72.4)	64.8 (57.2–71.7)	63.9 (55.7–71.4)
Invasive mechanical ventilation duration, days	3 (1–10)	2 (1–5)**	2 (1–5)
Severe bronchopulmonary dysplasia^3^	8.2 (5.7–11.7)	3.5 (1.6–7.7)***	4.0 (1.8–8.8)
Neonatal antibiotics >3 days	17.7 (13.1–23.5)	18.5 (12.4–26.6)	17.7 (11.3–26.6)
PDA^4^ treated (COI^5^ and/or surgery)	22.2 (18.0–27.0)	20.8 (15.4–27.5)	20.5 (14.7–27.8)
Necrotizing enterocolitis	5.1 (3.2–8.1)	5.2 (2.7–9.8)	4.9 (2.3–10.0)
Intraventricular haemorrhage^6^	29.3 (24.5–34.6)	29.4 (22.9–37.0)	28.5 (51.5–36.8)
Retinopathy of prematurity	1.7 (0.8–3.8)	1.8 (0.6–5.4)	2.1 (0.7–6.3)
Abnormal auditory screening	3.4 (1.9–6.0)	1.2 (0.3–4.7)*	0.7 (0.1–4.9)
**Developmental care**			
Skin-to-skin (kangaroo) care	70.1 (64.9–74.8)	78.5 (71.7–83.9)***	78.8 (71.5–84.6)
Parental feeding support^7^	51.2 (45.7–56.7)	56.1 (48.6–63.4)	56.8 (48.6–64.7)
Breastfed at discharge	43.6 (38.2–49.2)	55.7 (48.1–63.1)***	56.0 (47.7–63.9)
Individualized DC program^8^	11.2 (8.2–15.2)	15.1 (10.5–21.2)*	16.4 (11.2–23.2)

Data are weighted to take into account the different inclusion periods.

Data are means (standard deviation) or % (95% CI).

Invasive mechanical ventilation duration is presented as median, 25th percentile and 75th percentile for infants with invasive mechanical ventilation.

^1^Transfered to another institution in the first hours following birth;

^2^HMD: hyaline membrane disease;

^3^Defined as administration of oxygen for at least 28 days plus need for 30% or more supplementary oxygen and/or mechanical ventilatory support and/or continuous positive airway pressure at 36 weeks’ postmenstrual age;

^4^PDA: persistent ductus arteriosus;

^5^COI: cyclooxygenase inhibitor;

^6^Grade 1 or 2 intraventricular haemorrhage;

^7^Swaddling and/or sucking and/or skin-to-skin contact during a feed;

^8^DC: developmental care, i.e. NIDCAP or sensory motor program.

*p<0.05,

**p<0.01,

***p≤0.001 included *vs* non-included.

OLIMPE mothers who still participated in the study at 6 months corrected age had a lower risk of being probably depressed (as measured by the CES-D scale) and were less anxious (as measured by the STAI scale) than OLIMPE mothers who discontinued study participation.

### ADS scale components

At discharge, 43.3% of the i-ADS scales and 15.3% of the m-ADS scales had at least one unobserved component. Two unobserved items were particularly frequent in infants: touching (a) and touching (b), respectively 78.3% and 25.0%. For mothers, the most frequently unobserved items were touching (b) and holding, respectively 41.0% and 14.7%. An association was found between the observation (or not) of the items touching (a) in i-ADS and touching (b) in m-ADS (p<0.001).

At 6 months corrected age, the rates of unobserved components on the infant and mother subscales had decreased to 13.9% and 12.7%, respectively. All items on both scales were observed in more than 90% of the cases (Table 3).

**Table 3:** Frequency of unobserved items on ADS infant and mother subscales at discharge and 6 months.

	At discharge	At six months
	(N = 163)	(N = 148)
	n (%)	n (%)
**ADS infant subscale**		
Gazing	7 (4.4)	0 (0.0)
Vocalizing	2 (1.1)	0 (0.0)
Touching (a)	128 (78.3)	9 (5.9)
Touching (b)	41 (25.0)	7 (4.3)
Holding	20 (12.0)	9 (6.1)
Affect	4 (2.5)	0 (0.0)
Proximity	20 (12.2)	0 (0.0)
**ADS maternal subscale**		
Gazing	0 (0.0)	2 (1.4)
Vocalizing	0 (0.0)	0 (0.0)
Touching (a)	2 (1.3)	0 (0.0)
Touching (b)	66 (41.0)	10 (6.4)
Holding	24 (14.7)	9 (6.1)
Affect	1 (0.6)	0 (0.0)
Proximity	0 (0.0)	0 (0.0)

Data are weighted to take into account the different inclusion periods.

ADS infant subscale: touching (a): touches or reaches towards mother; touching (b): pulls away from mother’s touch.

ADS maternal subscale: touching (a): touches or reaches toward child; touching (b): pulls away from child’s touch.

### ADS scale interrater reliability

The ADS scale was independently assessed by two professionals in 59 dyads at discharge: 36.2% (59/163), and in 53 dyads at 6 months corrected age: 35.8% (53/148) (Table 4).

**Table 4:** Interrater reliability of ADS infant and mother subscales at discharge and 6 months.

	At discharge (n = 59)		At six months (n = 53)	
	Percentage of agreement	Weighted kappa (95% CI)	Percentage of agreement	Weighted kappa (95% CI)
**ADS infant subscale**				
Gazing	72.4	0.60 (0.42–0.78)	79.2	0.68 (0.49–0.86)
Vocalizing	72.4	0.59 (0.41–0.77)	75.5	0.65 (0.47–0.83)
Touching (a)	-	-	70.0	0.54 (0.32–0.75)
Touching (b)	60.5	0.40 (0.14–0.65)	70.6	0.44 (0.19–0.68)
Holding	77.6	0.53 (0.29–0.78)	79.2	0.50 (0.17–0.84)
Affect	73.7	0.39 (0.12–0.66)	69.8	0.51 (0.30–0.71)
Proximity	72.2	0.62 (0.45–0.80)	75.5	0.62 (0.42–0.82)
Disordered i-ADS	78.0	0.56 (0.34–0.78)	84.9	0.63 (0.38–0.88)
**ADS maternal subscale**				
Gazing	59.3	0.50 (0.33–0.67)	71.1	0.48 (0.24–0.71)
Vocalizing	78.0	0.76 (0.64–0.88)	71.7	0.63 (0.45–0.81)
Touching (a)	72.4	0.73 (0.60–0.86)	81.1	0.69 (0.50–0.88)
Touching (b)	-	-	70.8	0.39 (0.16–0.62)
Holding	66.0	0.51 (0.29–0.73)	66.7	0.21 (-0.05–0.47)
Affect	58.6	0.43 (0.22–0.65)	58.5	0.45 (0.25–0.64)
Proximity	74.6	0.71 (0.58–0.85)	71.7	0.56 (0.35–0.76)
Disordered m-ADS	83.0	0.66 (0.47–0.86)	79.2	0.53 (0.28–0.79)

Reliability analyses do not take into account the survey weights.

Given the high rate of unobserved components, reliability was not studied for "touching (a)" on the infant subscale and "touching (b)" on the mother subscale at discharge.

ADS infant subscale: touching (a): touches or reaches towards mother; touching (b): pulls away from mother’s touch.

ADS maternal subscale: touching (a): touches or reaches toward child; touching (b): pulls away from child’s touch.

At discharge, the percentages of agreement varied between 60.5% (touching (b)) and 77.6% (holding) for infants and between 58.6% (affect) and 78.0% (vocalizing) for mothers (unweighted percentages), while weighted Cohen’s kappa coefficients extended from 0.39 (affect) to 0.62 (proximity) for infant items, and from 0.43 (affect) to 0.76 (vocalizing) for maternal items.

At 6 months, the percentage of agreement and the weighted kappa coefficients for infant items were slightly higher compared with those at discharge and slightly lower for maternal items.

Interrater reliability for the presence or absence of an interaction disorder was determined for each subscale. Reliability was satisfactory for m-ADS at discharge [kappa: 0.66 (0.47–0.86)] and i-ADS at 6 months [kappa: 0.63 (0.38–0.88)], and moderate for i-ADS at discharge [kappa: 0.56 (0.34–0.78)] and m-ADS at 6 months [kappa: 0.53 (0.28–0.79)].

### Mother-infant interaction disorders

Due to the high rate of unobserved components on "touching (a)" on the infant subscale and "touching (b)" on the mother subscale at discharge, these items were not taken into account in classifying the mother-infant interaction disorders at discharge.

#### At discharge

At discharge (n = 163), interaction based on m-ADS was classified as disordered in 48.6 [40.9–56.4]%, including 49.8 [38.7–61.0]% resistant behaviour, 46.2 [35.3–57.5]% avoidant behaviour, and 3.9 [1.2–11.8]% combining resistant and avoidant behaviours. Based on i-ADS, interaction was classified as disordered in 36.5 [29.3–44.2]%, including 17.4 [9.5–29.8]% resistant behaviour, 72.2 [58.9–82.4]% avoidant behaviour, and 10.4 [4.6–21.8]% combined insecure behaviours.

Thus, 35.9 [28.8–43.7]% of the dyads had no disorder of interactions on either of the 2 sub-scales, 43.5 [36.0–51.4]% had an interaction disorder observed on one of the two subscales, and 20.6 [15.0–27.6]% an interaction disorder observed on the 2 sub-scales.

#### At six months

At 6 months (n = 148), m-ADS suggested altered interaction in 32.6 [25.4–40.7]%, including 40.1 [26.8–55.0]% resistant behaviour, 49.2 [34.9–63.5]% avoidant behaviour and 10.7 [4.4–23.9]% combining resistant and avoidant behaviours. Altered interaction was observed in 26.0 [19.4–33.8]% of i-ADS, with 18.8 [8.9–35.4]% resistant, 73.1 [56.1–85.3]% avoidant and 8.0 [2.5–23.2]% combined insecure behaviours.

Thus, 57.8% [49.6–65.6]% of the dyads had no disorder of interactions on either of the 2 sub-scales, 25.8 [19.3–33.6]% had an interaction disorder observed on one of the two subscales, and 16.4 [11.2–23.4]% an interaction disorder observed on the 2 sub-scales.

Disordered interactions on i-ADS significantly decreased between discharge and 6 months (p<0.001, McNemar’s test for paired data).

At 6 months, infant and mother disordered interactions based on i-ADS and m-ADS were significantly associated (p<0.001). Interaction disorders based on i-ADS were also associated with the previous observation of interaction disorders at discharge on both subscales (Table 5).

**Table 5:** Relationships between disordered interaction at discharge and 6 months based on ADS infant (i-ADS) and mother (m-ADS) subscales.

	Disordered i-ADS at discharge		Disordered m-ADS at discharge		Disordered i-ADS at 6 months		Disordered m-ADS at 6 months	
	Yes	No	Yes	No	Yes	No	Yes	No
Proportion of disordered i-ADS at 6 months	36.6 (24.3–51.1)	20.9 (13.5–30.9)	34.4 (24.1–46.5)	18.7 (10.9–30.2)	NA		50.3 (36.3–64.2)	14.2 (8.5–22.7)
p-value	<0.001 ^MN^		0.043				<0.001	
Proportion of disordered m-ADS at 6 months	52.3 (38.3–66.0)	19.7 (12.6–29.6)	37.4 (26.7–49.5)	25.7 (16.5–37.7)	63.1 (46.7–76.9)	21.9 (15.0–30.7)	NA	
p-value	<0.001		0.419 ^MN^		<0.001			

Data are % (95 CI %).

NA: not appropriate.

p-values from Pearson’s Chi-square tests.

^MN^ p-values from McNemar’s test for paired data.

### Association between maternal emotional state and mother-infant interaction disorders

Table 6 shows the proportions of interaction disorders in mothers and children at discharge and 6 months corrected age, according to the maternal mental health evaluations (CES-D, STAI, and mPPQ scales). No association was found between maternal depression, anxiety, or stress and interaction disorders based on the infant and mother subscales of the ADS.

**Table 6:** Disordered interaction at discharge and 6 months: Relationships with maternal emotional state and infant social withdrawal behaviour.

	n	m-ADS^1^, at discharge		m-ADS^1^, at 6 months		i-ADS^2^, at discharge		i-ADS^2^, at 6 months	
	%	% (95% CI)	p-value	% (95% CI)	p-value	% (95% CI)	p-value	% (95% CI)	p-value
**Maternal emotional state**									
Depressive symptoms (CES-D) at discharge	n = 155		0.645		0.419		0.833		0.682
Not at risk (score <16)	61.5	50.8 (40.5–61.1)		30.4 (21.5–41.0)		34.5 (25.3–45.0)		22.0 (14.4–32.1)	
Possible (score 16–22)	18.6	40.2 (23.0–60.2)		29.0 (14.3–50.0)		39.2 (22.2–59.2)		25.8 (12.0–47.0)	
Probable (score ≥23)	19.9	49.0 (31.2–67.1)		44.3 (25.6–64.8)		39.8 (23.5–58.8)		30.7 (15.2–52.2)	
Anxiety (STAI-State) at discharge	n = 154		0.972		0.297		0.640		0.354
Not significant (score ≤40)	74.9	48.9 (39.4–58.5)		30.1 (21.9–39.8)		35.5 (26.9–45.1)		23.1 (15.9–32.4)	
Significant (score >40)	25.1	49.3 (33.3–65.3)		40.4 (24.4–58.7)		39.9 (25.2–56.7)		31.6 (17.3–50.5)	
Anxiety (STAI-Trait) at discharge	n = 157		0.273		0.684		0.226		0.236
Not significant (score ≤40)	67.3	44.8 (35.1–55.0)		32.4 (23.7–42.5)		32.5 (23.8–42.6)		22.6 (15.3–32.1)	
Significant (score >40)	32.7	54.5 (40.4–68.0)		36.1 (22.4–52.5)		42.8 (29.6–57.1)		32.7 (19.5–49.3)	
Depressive symptoms (CES-D) at 6 months	n = 134	NA			0.796	NA			0.697
Not at risk (score <16)	81.6			32.8 (24.5–42.3)				26.1 (18.6–35.3)	
Possible (score 16–22)	8.0			28.6 (9.5–60.6)				19.1 (4.8–52.6)	
Probable (score ≥23)	10.4			41.3 (18.0–69.3)				34.8 (13.7–64.3)	
Anxiety (STAI-State) at 6 months	n = 133	NA			0.977	NA			0.577
Not significant (score ≤40)	86.5			32.7 (24.6–41.9)				25.6 (18.3–34.6)	
Significant (score >40)	13.5			32.3 (14.0–58.2)				32.3 (14.0–58.2)	
Anxiety (STAI-Trait) at 6 months	n = 133	NA			0.803	NA			0.200
Not significant (score ≤40)	74.4			32.8 (24.1–42.9)				23.5 (16.1–33.1)	
Significant (score >40)	25.6			35.2 (20.6–53.2)				35.2 (20.6–53.2)	
Post-traumatic stress disorders (mPPQ) at 6 months	n = 133	NA			0.298	NA			0.729
No (score <19)	54.3			37.5 (26.8–49.6)				26.0 (16.9–37.8)	
Yes (score ≥19)	45.7			28.8 (18.6–41.6)				28.8 (18.6–41.6)	
**Infant social withdrawal**									
Low social behavior (ADBB) at 6 months	n = 146	NA			0.179	NA			0.002
No (score <5)	87.8			30.4 (23.0–39.0)				21.3 (15.0–29.4)	
Yes (score ≥5)	12.2			46.4 (25.3–69.0)				56.6 (33.6–77.0)	

Data are % (95% CI).

^1^Disordered interaction based on Attachment During Stress (ADS) mother subscale (m-ADS);

^2^Disordered interaction based on Attachment During Stress (ADS) infant subscale (i-ADS); CES-D: Center for Epidemiologic Studies Depression Scale; STAI: State Trait Anxiety Inventory for adults (STAI-State: respondent feelings "right now"; STAI-Trait: respondent feelings "generally"); mPPQ: modified Perinatal Posttraumatic Stress Disorder Questionnaire; ADBB: Alarm Distress Baby Scale; NA: not appropriate.

### Association between infant social withdrawal behaviour and mother-infant interaction disorders

The infant social withdrawal behaviour measured by the ADBB scale was strongly associated with altered interactions on the ADS infant (p = 0.002) but not mother (p = 0.179) subscale (Table 6).

### Association between infant family-centred care and mother-infant interaction disorders

No association was found between the NICUs’ policies regarding KC or BF and disordered interaction at discharge and at 6 months (data not shown).

## Discussion

This study showed that perinatal care professionals were able to assess the early interactions between mothers and their very preterm infants with acceptable reliability after a period of training. Insecure attachment behaviours at 6 months infant corrected age were associated with previous identification at discharge. The behaviours in the mothers were anticipated by the observation of disordered infant interactions, and in the infants by the non-optimal interactional patterns of both the mothers and their infants. Also, insecure infant attachment at 6 months corrected age was not related to maternal mental health but was strongly associated with infant social withdrawal behaviour.

To our knowledge, OLIMPE is the first large cohort of very premature neonates in which early mother-infant interactions were studied just before discharge from the NICU. We selected a scale that could be scored during or just after live observation and rapidly coded [22]. Although the ADS scale is compatible with self-guided training, two one-day meetings were organized to ensure standardized procedures in all centres, especially for item scoring, and a written protocol summarizing all instructions was distributed before the start of the study. Despite the containment provided during the examination to promote interactions, the infants’ immature motor skills were a limiting factor in the observation of "touching" items on the infant subscale at discharge, and thus the corresponding maternal reaction, "touching (b)". Although previous studies have not reported this restriction in scale use, it should be noted that they included only term infants of at least 3 months old, rather than very premature neonates examined near term [25, 36]. Theoretically, the CARE-Index also assesses the quality of interaction and dyadic characteristics from birth to 15 months [37]. However, its performance in the context of premature infants having less than 6 months corrected age has never been explored [13, 20]. In addition, this tool requires extensive training, systematic videotaping of the interactions, and experienced coders [22]. Censullo’s dyadic mutuality code (DMC) is another instrument that can be used in the first 6 months of life to specifically explore synchronicity in the dyad [38], but its sensitivity has been questioned in a context quite comparable to ours [39].

The literature indicates that different procedures have been followed to interpret ADS scoring. Hale et al. [25] averaged the mean scores of the seven items, using the subscale scores. We nevertheless found this scoring method to be inappropriate for detecting insecure behaviours, which are positioned at the extremes of the 5-point scale. We therefore based our rating on the occurrence of insecure behaviours determined independently from the infant (i-ADS) and mother (m-ADS) subscales, as did Carcamo et al. [36]. However, we did not use their classification which prevented the coding of a substantial number of scales. Thus, we considered that the observation of at least one typical behaviour of insecure attachment or two rather avoidant behaviours was enough to confirm a disordered interaction, which allowed us to settle all cases. The rate of nearly 30% for insecure behaviours on both the maternal and infant subscales at 6 months was fairly consistent with the controlling pattern of interaction observed in 28% of mother-infant dyads using the CARE-Index at the same age [25, 36]. Moreover, in a cohort of 117 children born before 32 weeks of gestation or below 1500 g, a recent study observed 12.8% avoidant and 23.1% resistant/ambivalent attachment at a corrected age of 2 years using the gold standard: the strange situation procedure [40].

Our observation that a disordered maternal interaction at discharge may anticipate a disordered infant interaction at 6 months was consistent with the transactional theory of development, which proposes that non-optimal interactional patterns exacerbate already disorganized infant behaviour [41]. Intervention programs based on this theory and begun during NICU hospitalization therefore may improve infant temperament, mother-infant interactions and parenting stress in the early postnatal years [42].

Most of the research on parental mental health following very preterm birth has focused on maternal depression, with rates of clinically significant depression ranging between 15% and 40% in the first postnatal year, compared with approximately 10% after term delivery [43]. Despite the documented adverse effects of postpartum depression on mother-infant interactions, we found no association between the CES-D and ADS scales. This result was not explained by maternal antidepressant treatment, which might have potentially decreased the impact of depression on these interactions, as mothers suffering from a psychiatric disorder, including severe depression requiring treatment, were excluded from the study. CES-D screens for women at risk for depression, but it cannot be used to diagnose major depression. Furthermore, the impact of early maternal depression on attachment security is statistically low, with the magnitude of effect extremely variable from one study to another [44]. Maternal anxiety has been associated with less touching, speaking and responsiveness to the premature infant during NICU hospitalization, which may decrease the infant's engagement in interaction [45]. However, the studies that have assessed these early interactions found that anxiety had inconsistent effects depending on its intensity, duration, co-occurrence with depression, and the tools used to measure it [12, 18, 39]. Attention is increasingly being focused on the symptoms of maternal posttraumatic stress, which is experienced by 35–45% of the mothers of premature infants at 6 months infant age, as noted in the OLIMPE study as in others [19, 20, 46]. Yet to date, very few studies have examined the influence of maternal traumatic reaction on the quality of mother-infant interaction at 6 months. Consistent with our results, Muller-Nix et al. found no significant difference in maternal and infant interactional behaviours [19]. However, maternal stress influenced the dyadic interactive pattern, with more "controlling mother-compliant infant" dyads [20].

A robust association was found at 6 months between disordered interaction on the ADS infant subscale and infant social withdrawal behaviour. In our opinion, this finding adds external validity to the results of the ADS scale, as the ADBB scale includes elements of observation in relationship to the observer rated outside the mother-infant interaction sequences. Indeed, withdrawal has been interpreted as a way for the infant to deal with a lack of synchrony in the parent-infant relationship, and the association between early mother-infant interaction and later social withdrawal has been suggested [47]. Our rate of withdrawal was nevertheless difficult to interpret, in part because the OLIMPE study is the first to provide such data in very preterm neonates. A study performed in our country found a prevalence of 14% at 12 months and a clear association with low birth weight and preterm birth [21].

The ADS scale results suggested that, for family-centred care, the centres’ policies regarding KC or BF did not seem to influence the occurrence of disordered interactions at discharge and at 6 months. Although policies supporting neurodevelopmental care have progressed in our country [27], few children of the EPIPAGE 2 and OLIMPE cohorts were enrolled in an individualized developmental care program with a family-centred approach, yet these programs may well increase parental self-confidence and competence and promote a positive interactional style.

### Strengths and limitations

The OLIMPE study drew from a representative national cohort, EPIPAGE 2, but we observed only marginal differences in maternal characteristics between the included and non-included populations. Notably, socioeconomic conditions, pregnancy care and complications, and delivery context were comparable. The lower rate of in-utero transfer suggested that the OLIMPE mothers dwelled preferentially in urban or suburban areas endowed with a level 3 perinatal unit. OLIMPE mothers were less frequently hospitalized during pregnancy, although it should be noted that hospitalization during pregnancy has not been correlated with the quality of interactions in the dyads [39]. These mothers also more frequently breastfed their infants at discharge but, again, a direct relationship has not been demonstrated between breastfeeding practice and interaction quality at 6 months or attachment security at 12 months [48].

On the other hand, the OLIMPE neonates may be considered at lower risk for interaction disorders compared with the neonates in the EPIPAGE 2 study. Given the objective of the OLIMPE study, i.e. to individualize the specific effect of prematurity on these interactions and their disorders, we excluded particular circumstances or serious complications associated with prematurity. This selection may have led to an underestimation of disordered interactions at discharge and at 6 months. Interrater reliability could only be studied on a fraction of the sample, i.e. 36.2% of the dyads observed at discharge and 35.8% at 6 months. The reliability in the assessments of interaction disorder on the mother and infant subscales was lower than that reported by others [25, 36], but our study is nevertheless the first to assess reliability in real life, and not *a posteriori* on videotapes. Only preterm singletons were assessed, because the simultaneous presence of two professionals trained to observe interactions could not be ensured in all centres. Premature twins nevertheless appear to be at greater risk for poorer quality of maternal interactions [49].

## Conclusions

This prospective multicentre observational cohort study confirmed the high prevalence of disordered mother-infant interactions and maternal distress in the few months following very preterm birth. We do not as yet have long-term outcomes for the OLIMPE infant study, but neurodevelopmental outcome will be assessed on the basis of the difficulties observed in the early interactions. Parents and the environment can modulate the effects of biological factors on child development. Our work suggests that the ADS scale can help to target those dyads that would most benefit from early hospital- and home-intervention programs designed to improve parenting behaviours, the quality of the home environment, and the outcomes for children and their families [50].
